# Influence of intensive rearing, continuous and rotational grazing systems of management on parasitic load of lambs

**DOI:** 10.14202/vetworld.2019.1188-1194

**Published:** 2019-08-07

**Authors:** M. S. Ram Prasad, S. Meenakshi Sundaram, P. Tensingh Gnanaraj, C. Bandeswaran, T. J. Harikrishnan, T. Sivakumar, P. Azhahiannambi

**Affiliations:** 1 Department of Livestock Production and Management, Madras Veterinary College, Chennai, Tamil Nadu, India; 2 Instructional Livestock Farm Complex, Tamil Nadu Veterinary, and Animal Sciences University, Chennai, Tamil Nadu, India; 3 Department of Livestock Production and Management, Tamil Nadu Veterinary and Animal Sciences University, Chennai, Tamil Nadu, India; 4 Department of Animal Nutrition, Veterinary College and Research Institute, Thanjavur, Tamil Nadu, India; 5 Department of Veterinary Parasitology, Tamil Nadu Veterinary and Animal Sciences University, Chennai, Tamil Nadu, India; 6 Department of Animal Nutrition, Veterinary College and Research Institute, Thanjavur, Tamil Nadu, India; 7 Department of Veterinary Parasitology, Madras Veterinary College, Tamil Nadu Veterinary and Animal Sciences University, Chennai, Tamil Nadu, India

**Keywords:** ewe lambs, FAMACHA^©^ scores, grazing systems, parasitic load

## Abstract

**Aim:**

A trial was conducted to assess the influence of parasitic load on the lambs reared under the intensive system, continuous grazing, and rotational grazing systems of management.

**Materials and Methods:**

A total of thirty numbers of the undetermined breed of ewe lambs around 4-5 months of age were randomly selected and allotted to three treatment groups: T_1_ (intensive system – control), T_2_ (rotational grazing), and T_3_ (continuous grazing). The T_1_ group lambs were raised under a stall-fed system of management, the T_2_ group lambs were grazed under rotational grazing strategy in four paddocks of plot-A, while the T_3_ group lambs were continuously grazed in plot-B.

**Results:**

At the end of the study, there was a highly significant difference (p=0.01) in the fortnightly strongyle egg count per gram (EPG) of feces among the lambs pertaining to the three treatment groups; the lambs in T_3_ had a higher strongyle EPG compared to T_2_ lambs. With regard to the overall reduction in EPG from the initial count, lambs under rotational grazing showed the maximum decrease of 54.52% compared to lambs under T_3_ (continuous grazing). There was a strong positive correlation noticed between the mean temperature of the day at each fortnight and the subsequent EPG at each fortnight with R^2^=0.87. There was a strong positive correlation noticed between mean FAMACHA^©^ scores and the EPG with R^2^=0.84, R^2^=0.83, and R^2^=0.83 for T_1_, T_2_, and T_3_, respectively.

**Conclusion:**

The grazing management with pasture rotation should be considered as a viable option for sustainable parasitic control in case of grazing-dependent livestock husbandry in India.

## Introduction

India possesses 65.06 million sheep (19^th^ Livestock Census - All India Report, 2012). The multifaceted sheep husbandry forms an integral part of the rural agrarian economy of arid and semi-arid zones of India [1].

Under Indian conditions, majority of sheep-raising has been grazing dependent. The practice of shepherding and grazing has been the core fabric of sheep husbandry in India. However, this practice of grazing sheep suffers from threatening drawbacks such as poor quality of grazing land and the worm burden which hampers the production. The parasite burden in rangeland sheep and other livestock has been a major concern for the Indian livestock sector. The parasites play a major part in contributing to the production losses of sheep husbandry in India. In India, *Haemonchus contortus* is the most pathogenic nematode of sheep and goat [2,3], which is completely pasture borne. Overuse of anthelmintics has raised the problem of drug resistance and food security [4-7]. Apart from chemotherapeutic intervention, a sustainable helminth control strategy is required for grazing-dependent livestock husbandry of India.

The lack of awareness among the rural farmers, shepherds about proper grazing management is one of the major reasons for pasture borne-helminthiasis in sheep husbandry. Grazing management with pasture rotation plays a major part in sustainable helminth control in livestock [8-10].

In the present study, the concept of rotational and continuous grazing was adopted in developed pasture with the composition of *Panicum* *maximum* (Guinea grass), *Stylosanthes* spp., *Desmanthus* spp., and *Cenchrus* *ciliaris* (buffelgrass), and their influence over the worm burden egg count per gram (EPG) of feces of the animals was studied and compared with the intensive system of management, hoping that it would pave the way for the solutions for the issues discussed earlier.

## Materials and Methods

### Ethical approval

No invasive methods were used. Experiment was carried out at University Research Farm. The animals were maintained under standard managemental conditions without any unnecessary stress or harm.

### Experimental location

The experiment was carried out at University Research Farm, Tamil Nadu Veterinary and Animal Sciences University, Chennai-51, Tamil Nadu, India. It lies between latitudes 12° 9’ and 13° 9’ and longitudes 80° 12’ and 80° 19’ E with an altitude of 22 m above sea level. The experiment was conducted for 5 months from January 2016 to May 2016. The maximum and minimum temperatures recorded during the experimental period were 36.64°C and 30.51°C, respectively. Rainfall was observed in January (185.55 mm) and May 2016 (0.95 mm).

### Animals and pasture management

The trial was conducted with thirty lambs of the undetermined breed in the age group of 4-5 months maintained at the University Research Farm, Madhavaram. The experimental animals were dewormed 1 month before the start of the experiment using ivermectin (NEOMEC^®^ - INTAS Pharmaceuticals, India) at 200 mcg/kg body weight subcutaneously.

Ten animals for each treatment were randomly allotted based on body weight to the following three treatment groups: T_1_ (intensive system – control), T_2_ (rotational grazing), and T_3_ (continuous grazing). The T_1_ group lambs were raised under a stall-fed system of management and were sheltered in pens on the slatted floor made of wooden slats of 4-cm width. The gap between the slats was 18 mm. The slatted floor was elevated 2 feet from the ground level. The animals were fed with *P. maximum* (Guinea grass), *Stylosanthes* spp., *Desmanthus* spp., and *C. ciliaris* (buffelgrass) harvested from both the grazing plots A and B. The succulent and legume fodders were fed in the ratio of 1:1.

A pasture of 0.18 hectare was developed with *P. maximum* (Guinea grass), *Stylosanthes* spp., *Desmanthus* spp., and *C. ciliaris* (buffelgrass) for the lambs under grazing systems. The lambs after grazing were kept in night shelters. All the experimental animals were supplemented with 100 g of concentrate feed per day.

### Grazing management

The T_2_ group lambs were grazed under rotational grazing strategy in the four paddocks (2500 sq. feet each) of plot-A, while T_3_ group lambs were continuously grazed in plot-B (10,000 sq. feet). The grazing period was 4-5 days in each paddock since the infective stage of *H. contortus* L_3_ takes 7 days to develop [11].

The rest period was 15 days per paddock since the fodder biomass was optimum at this stage. A rest period more than 15 days was not practically applicable as the biomass was surplus at that stage. Further, Guinea grass after 15 days of the rest period had a sward length >15 cm which makes the parasite prevalence less [9] in the top surface of the sward to be consumed by the lambs.

The T_3_ group lambs were allowed for continuous grazing in the plot-B. Both T_2_ and T_3_ group lambs were allowed to graze for 6-8 h per day from 7 am to 5 pm.

### Experimental sampling and analysis

#### Parasitic load

The fecal examination was done by simple floatation method using a saturated sodium chloride solution, and the eggs were enumerated using the Modified McMaster technique [12-14].

#### Identification of larvae

Coproculture using Harada-Mori culture method [15] was performed, and then, the larvae were identified by studying the morphological features [16].

#### FAMACHA^©^ score analysis

The FAMACHA^©^ chart (FAffa and Malan Chart) was prepared by South African Researchers Dr. Francois Malan, Gareth Baeth and Jan Van Wyk. It is used to interpret the degree of anemia through a color guided chart, comparing it with the color of conjunctival mucous membrane and subsequently treating the animals.

The parasitic burden in animals was monitored using the FAMACHA^©^ eye color chart. The animals were given scores based on the color of their conjunctiva. The color of conjunctiva was classified into five categories according to the FAMACHA^©^ eye color chart, from red - very good (1), through red-pink - good (2), pink - needs deworming (3), pink white - anemic (4) and white - highly anemic (5) [17].

### Statistical analysis

The data collected were subjected to the statistical analysis using ANOVA, t-test, coefficient of determination (r^2^), and Kruskal–Wallis test – nonparametric test [18] using SPSS Inc. software Version 20 (IBM, USA).

## Results

### Fortnightly strongyle EPG of the three treatment groups

Post-initial deworming, the mean EPG of the lambs under all the treatment groups before the start of the trial was very minimum <100 EPG.

At the end of the trial, there was a highly significant difference (p=0.01) in the fortnightly strongyle EPG in the lambs pertaining to the three treatment groups as seen in Table-1. The T_3_ lambs had a higher strongyle EPG compared to T_2_ lambs while the lowest EPG was observed in the lambs of the T_1_ treatment group.

**Table 1:** The mean±SE and analysis of variance of fortnightly strongyle egg count per gram in lambs of three treatment groups.

Fortnights	T_1_ (intensive system)	T_2_ (rotational grazing)	T_3_ (continuous grazing)	F-value
1	1860^c^±0155.00	4310^b^±1173.0	7170^a^±701.9	11.94**
2	2700^b^±0614.00	3500^b^±967.0	7480^a^±788.0	10.16*
3	2270^b^±1004.00	3020^b^±710.0	7280^a^±617.0	11.17**
4	2840^b^±0854.00	3260^b^±791.0	6970^a^±817.0	8.74**
5	2810^b^±0838.70	3390^b^±797.1	6943^a^±817.0	7.40**
6	1760^b^±0337.00	3850^a^±645.0	4570^a^±508.0	8.09**
7	1230^b^±0289.00	3130^a^±459.0	4380^a^±789.1	8.22**
8	1250^b^±0216.10	2220^b^±562.8	4150^a^±897.0	5.59**
9	1120^a^±0191.30	1980^ab^±246.0	3760^a^±1098.0	4.17*
10	1260^a^±0224.00	1960^ab^±317.0	3700^a^±983.0	4.23*

* Significant at 5% level (p<0.05).

** Significant at 1% level (p<0.01). Means bearing different superscript in the same column differ significantly

### Overall percentage of reduction in EPG from the first fortnight in lambs across all treatment groups

**Figure-1:**
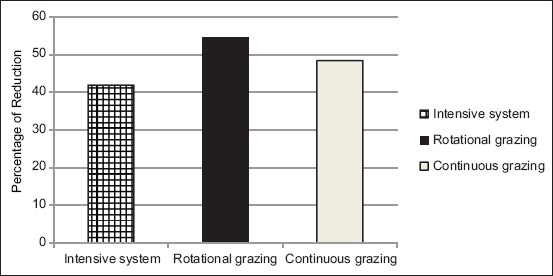
Overall percentage of reduction in different treatment groups.

With regard to an overall reduction in EPG from the first fortnight, the lambs under rotational grazing showed the maximum decrease of 54.52% followed by continuous grazing with a decrease of 48.3% and the intensive system with a decrease of 41.9%. The overall percentage of reduction in the mean EPG of three treatment groups is shown in Figure-1.

### The percentage of reduction in EPG between first and subsequent fortnights in the lambs under different treatment groups

The percentage of reduction in EPG between first and subsequent fortnights in lambs under different treatment groups is given in Table-2.

**Table 2:** Percentage of reduction between the first and subsequent fortnights.

Treatment	Percentage of reduction between first versus subsequent fortnights								

	1 versus 2	1 versus 3	1 versus 4	1 versus 5	1 versus 6	1 versus 7	1 versus 8	1 versus 9	1 versus 10
T_1_	−32.53	1.40	−20.36	−60.65	11.047	37.96	37.60	39.84	36.84
T_2_	−70.71	−58.23	−14.44	−36.53	−45.62	−8.07	19.22	50.44	44.14
T_3_	−11.90	−11.55	−8.049	3.034	27.00	31.66	33.70	50.44	44.15

### Difference in EPG between the initial and the subsequent fortnights of the three treatment groups

In case of difference in EPG between initial and the subsequent fortnights within the treatment groups, lambs in T_3_ under continuous grazing had a significant (p=0.01) difference from the sixth fortnight onward. Although T_2_ lambs under rotational grazing did not show any significant difference in mean EPG between initial and the subsequent fortnights, there was a significant difference observed at p=0.06 from the penultimate fortnight. Lambs in T_1_ under intensive system had a significant (p=0.01) difference from the seventh fortnight onward. The analysis of variance of difference in EPG between the initial and the subsequent fortnights is given in Table-3.

**Table 3:** Analysis of variance of difference in mean egg count per gram between the initial and subsequent fortnight.

Treatment	p-value - initial fortnight versus subsequent fortnights								

	1 versus 2	1 versus 3	1 versus 4	1 versus 5	1 versus 6	1 versus 7	1 versus 8	1 versus 9	1 versus 10
T_1_	0.570	0.890	0.640	0.340	0.330	0.038**	0.012**	0.050**	0.016**
T_2_	0.601	0.359	0468	0.525	0.361	0.126	0.068	0.068	0.069
T_3_	0.770	0.910	0.836	0.836	0.008**	0.017**	0.016**	0.018**	0.010**

*Significant at 5% level (p<0.05).

** Significant at 1% level (p<0.01). Means bearing different superscript in the same column differ significantly

### Correlation between the mean temperature of the day at each fortnight and the subsequent EPG at each fortnight

There was a strong positive correlation noticed between the mean temperature of the day at each fortnight and the subsequent mean EPG at each fortnight with R^2^=0.87; there was also a similar trend noticed in each treatment groups with R^2^=0.57, R^2^=0.69, and R^2^=0.83 for T_1_, T_2_, and T_3_, respectively.

### Fecal culture – Harada-Mori culture method

The infective third-stage larvae (L_3_) of *H. contortus* were predominantly found along with other strongyloid larvae in the larval suspension retrieved from the Harada-Mori method. The infective third-stage larvae (L_3_) of *H. contortus* had the following morphological features: kink in tail sheath just posterior to tail; tail sheath endings into a fine whip-like filament.

### FAMACHA^©^ scores

A highly significant difference (p≤0.01) between the different treatment groups in FAMACHA^©^ scores was observed during January, February, and March. However, the FAMACHA^©^ scores remained the same during April and May. The FAMACHA^©^ scores of lambs and analysis of variance of the same are given in Table-4 using the non-parametric method, Kruskal–Wallis test.

**Table 4:** Monthly mean±SE and Kruskal–Wallis H-test analysis of FAMACHA^©^ scores of lambs under different treatment groups.

Treatment groups	Months				

	January	February	March	April	May
T_1_	3.0±0.15	3.0±0.15	2.9±0.15	2.8±0.15	2.7±0.15
T_2_	3.5±0.15	3.5±0.15	3.5±0.15	3.4±0.15	3.2±0.15
T_3_	4.1±0.12	4.1±0.14	4.0±0.14	3.7±0.14	3.2±0.14
p-value	0.003**	0.003**	0.009**	0.064^NS^	0.121^NS^

*Significant at 5% level (p<0.05).

** Significant at 1% level (p<0.01). Means bearing different superscript in the same column differ significantly

There was a strong positive correlation noticed between FAMACHA^©^ scores and the fecal egg count (FEC) with R^2^=0.84, R^2^=0.83, and R^2^=0.83 for T_1_, T_2_, and T_3_, respectively.

## Discussion

In this experiment, the influence of grazing strategies on the parasitic load of the lambs in a developed pasture has been studied for the first time in tropical conditions of Tamil Nadu, India.

The pasture-borne parasite nemeses have been a major concern for Indian pastoral animal husbandry. The anthelminthic treatment has not completely satisfied the need and lately repeated use had developed drug resistance among the helminths. Grazing management with pasture rotation plays a major part in sustainable helminth control in livestock as reported by Kumar *et al*. [9] and Waller [10]. Hence, grazing management with the strategy of rotational grazing and its influence on the parasitic load is studied.

1 month before the start of the trial, the experimental animals were dewormed using NEOMEC^®^ (ivermectin) at 200 mcg/kg body weight subcutaneously before 1 month of the start of the trial. Post-initial deworming, the mean EPG of the lambs under all the treatment groups before the start of the trial was very minimum <100 EPG.

At the end of the trial, the strongyle EPG differed significantly (p≤0.01) between the lambs pertaining to different treatment groups as seen in Table-1; further, the lambs under rotational grazing system of management had significantly higher reduction percentage of 54.52% in EPG from the initial fortnight than those under continuous grazing system (48.30%) of management.

The reason being is the majority of the parasite larvae (80%) lives in the first 5 cm of the vegetation. The rest period of 15 days ensured that the sward height of grasses in the rotational system of grazing paddocks remained >20 cm at any given point of time. Hence, the rotationally grazed T_2_ lambs were exposed to lower interrupted larval challenge throughout the experimental period.

However, the sward height of grasses in the continuously grazed paddocks remained well below 10 cm due to confined and continuous grazing by the animals without any control over them. This might have influenced the higher EPG count in continuously grazed T_3_ lambs due to the uninterrupted available quantum of larval challenge from the short swards of forage in plot-B.

In concurrence with the results of the present study [9,11,19-21], it was also observed that grazing with pasture rotation plays an important role in the control of pasture-borne helminths by decreasing the worm load compared to the continuous system of grazing.

The lower initial EPG in lambs under T_2_ compared to T_3_ may be attributed to the reduced area of the paddock (2500 sq. feet) allotted to them and thus the quantum of larval challenge, while the lambs under T_3_ grazed continuously in undivided plot-B (10,000 sq. feet) at the start of the trial. A high spurt of EPG observed immediately after the first fortnight of the trial can be attributed to high showers of the rain (185.55 mm) recorded during the January month of the trial.

**Figure-2:**
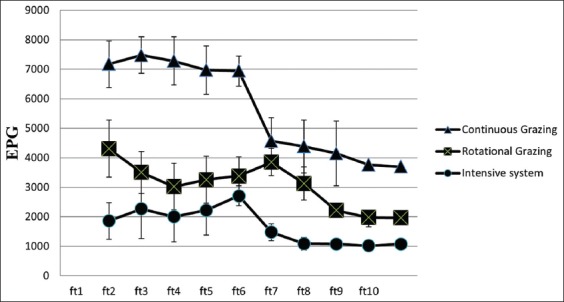
Mean±SE egg count per gram of lambs under the three treatment groups.

There was a linear decrease observed in the EPG in lambs under continuous grazing as seen in Figure-2. This may be attributed to the continuous larval challenge available for the animal in continuously grazed plot-B. The lambs might have been sensitized against the infection by acquiring immunity due to the continuously available larval challenge, which is also known as the self-cure phenomenon [22].

However, there was not a comparatively linear decrease in EPG of the lambs under rotational grazing as seen in Figure-2, since there was not any continuous larval challenge available for the animal since the lambs were rotated for every 5 days in the paddocks of plot-B has also a part to play as discussed earlier.

The animals belonging to T_1_ showed the presence of strongyle eggs in feces since they were fed with the fodder harvested from the grazing plots of T_2_ and T_3_. The lambs under T_1_-intensive system had the lowest EPG. There was a similar trend noticed in EPG of lambs in T_1_-like lambs in T_3_ over the experimental period because there were infective larvae present in the cut fodder and herbage fed to the lambs.

In case of the percentage of reduction in EPG between the first and subsequent fortnights, there was a linear decrease observed in the last few fortnights in lambs under T_3_ and T_2_, while there was a significant decrease (p≤0.01) in the EPG from sixth fortnight onward in continuously grazing lambs (T_3_). This might be attributed to the climatic factors – high mean temperature of the day and self-cure phenomenon developed due to continuous larval challenge available to the animal.

**Figure-3:**
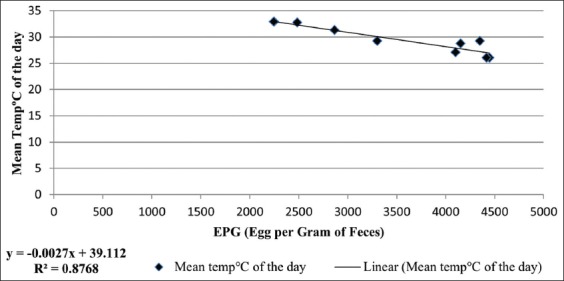
Correlation between the mean temperature of the day (°C) and the egg count per gram at each fortnight.

There was a strong positive correlation noticed between the mean temperature of the day at each fortnight and the subsequent EPG at each fortnight with R^2^=0.87 as seen in Figure-3, which infers that 87% of fluctuations at each interval of EPG is explained by the mean temperature of the day or vice versa. There was also a similar trend noticed in each treatment groups with R^2^=0.57, R^2^=0.69, and R^2^=0.83 for T_1_, T_2_, and T_3_, respectively. The intensive system - T_1_ had comparatively a lesser correlation coefficient R^2^=0.57 than the other treatment groups, which might be probably due to lesser mean temperature under the sheds than in the pastures.

The highest EPG in lambs pertaining to all the treatment groups during the experimental period was observed in January and February immediately after the northeast monsoon [23], and the EPG decreased drastically during the summer months of April and May where the mean temperature of the day was 32°C and the maximum temperature was up to 36°C [24,25].

The total number of mean egg count of the entire lot remained well below 3000 EPG throughout the experimental period; hence, they were not treated for the same [26]. The EPG of T_2_ lambs under rotational grazing remained well below the requisite of treatment (≤3000) throughout the experimental period, giving the practice of rotational grazing an edge over the continuous grazing strategy.

The infective third-stage larvae (L_3_) of *H. contortus* were predominantly found [2,16] along with other strongyloid larvae in the larval suspension retrieved from the Harada-Mori method. The infective third-stage larvae (L_3_) of *H. contortus* were identified by the morphological features: kink in tail sheath just posterior to tail; tail sheath ending into a fine whip-like filament [16].

Since the coproculture of the dung samples collected from the experimental animals revealed the presence of *H. contortus* larvae, the FAMACHA^©^ score analysis has been used to monitor the parasitic load.

**Figure-4:**
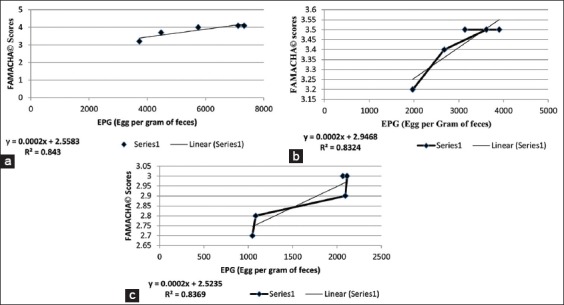
(a) Correlation between FAMACHA^©^ scores and egg count per gram (EPG) –continuous grazing; (b) Correlation between FAMACHA^©^ scores and EPG – rotational grazing; (c) Correlation between FAMACHA^©^ scores and EPG – intensive system.

The mean FAMACHA^©^ scores also differed significantly between the different treatment groups with the lambs under continuous grazing system scoring significantly (p≤0.01) higher FAMACHA^©^ scores as observed in Table-4. There was strong positive correlation noticed between FAMACHA^©^ scores and the FEC with R^2^=0.84, R^2^=0.83, and R^2^=0.83 for T_1_, T_2_, and T_3_, respectively, as seen in Figure-4, which infers that 84% of the change in FAMACHA^©^ scores is explained by the EPG or the vice versa. Hence, the scores of FAMACHA^©^ obtained via assessing the conjunctival mucous membrane was related to the EPG, more the count more was the conjunctival mucous membrane blanched, and the vice versa [26]. After the end of the experiment, all the animals were dewormed.

The grazing management with pasture rotation should be considered as a viable option for sustainable parasitic control in case of grazing-dependent livestock husbandry in India [2,6,27-30]. The shepherds should be educated on the exploitation of the grazing land by the practice of continuous grazing and should be advised to adopt rotational grazing strategies considering the fragility of the Indian pastures and the issues of anthelminthic resistance [8].

## Conclusion

To conclude, based on the results from the present study rotational grazing can be advocated to the farmers and the practice of continuous grazing to be discouraged as rotational grazing allows proper rest period for the pasture to replenish it selves and also ensures that the sward length of the pasture remains > 20 cm at any point time which minimizes the chances of L3 (Infective stage Larvae) getting into the host, as the latter remains till the sward length of only 5 cm. Hence the rotational grazing management can be a helping hand to reduce the economic losses inflicted due to pasture-borne helminthiasis in sheep husbandry with necessary steps taken by the Governmental and Non-Governmental organizations.

## Author’s Contributions

SMS and MSR designed and supervised the study. MSR analyzed samples. MSR drafted the manuscript. SMS, PTG, CB, TJH, TS, and PA discussed and edited the manuscript. All authors read and approved the final manuscript.
